# Deciphering the mechanism of women’s mental health: a perspective of urban–rural differences

**DOI:** 10.3389/fpubh.2025.1545640

**Published:** 2025-03-05

**Authors:** Changqin Chen, Ruying Chen, Qianhui Wang, Mengdi Zhang, Jinhui Song, Wen Zuo, Rong Wu

**Affiliations:** ^1^Guangzhou Urban Planning and Design Company Limited, Guangzhou, China; ^2^School of Architecture and Urban Planning, Guangdong University of Technology, Guangzhou, China

**Keywords:** women, mental health, livelihood capital, relative deprivation, urban–rural differences

## Abstract

**Background:**

Mental health accounts for a significant proportion of holistic health and affects women more significantly than men. Livelihood capital, defined as an indicator of these sources available for individuals or households to survive and develop, is a cost-effective field for ameliorating women’s mental health. However, the impact of these multiple factors of livelihood capital on mental health still requires further research Therefore, this study evaluates women’s mental health and investigates the correlation between livelihood capital (including human capital, physical capital, social capital, and financial capital) and women’s mental health.

**Methods:**

Based on the 2018 China Labor-force Dynamics Survey, this study explores the relationship and pathways between livelihood capital and women’s mental health, with the structural equation model. It also aimed to examine the impact of relative deprivation under the influence of livelihood capital on women’s mental health, focusing on urban–rural differences.

**Results:**

The results of this study are as follows: (1) Human capital, social capital and physical capital were positively correlated with women’s mental health, while financial capital showed a negative correlation; (2) Relative deprivation mediates the impact of livelihood capital on women’s mental health; (3) The impact of livelihood capital on women’s mental health is different between urban and rural regions. Urban women were more likely to be influenced by financial capital, whereas human capital, social capital and physical capital were key factors affecting rural women’s mental health. (4) Relative deprivation mediates the impact of livelihood capital on mental health in the rural sample, but not in the urban sample.

**Discussion:**

This study shows a complex relationship among livelihood capital, relative deprivation, and women’s mental health, with notable urban—rural differences. In rural areas, human, social, and physical capital positively affect women’s mental well—being. For example, better education reduces relative deprivation. Social support and improved housing also help. Conversely, financial capital has a negative link with mental health, more so in urban areas, likely due to urban pressure and the wealth—mental health relationship. Based on the findings, several actions are warranted. In social welfare, allocate more resources to rural areas for stronger women’s livelihood capital. Public services should improve rural housing and community integration. Expand and enhance mental health education for rural women. These steps can ease rural women’s mental health challenges and promote fairness in mental health outcomes.

## 1 Introduction

In recent years, with deeper understanding of gender, health, and well-being, women’s mental health has gradually become a focus within the international community. Mental health problems in women are serious and complex. According to data from the World Health Organization, women are more likely to suffer from mental disorders than men, such as depression, anxiety, and post-traumatic stress disorder (PTSD) (1). Evidence from the American Institute of Mental Health also shows that adult women have a significantly higher prevalence of mental illness (27.2%) than adult men (18.1%) (2). At the same time, this phenomenon is more prominent in low- and middle-income countries because of the imbalance between economic development needs, demographics, and cultural development (3). As a representative emerging economy and a country with a large population base, evidence shows that approximately 60% of mental health problems nationwide in China occur in women (4). Therefore, an exploration of women’s mental health in China is necessary. However, issues arising in relation to women’s mental health cannot only be attributed to specific gender characteristics, but also involve a complex interaction between personal, social, and structural pressures and vulnerabilities, that is, such issues are a product of gender socialization interaction (5). The social interaction process for women involves two main aspects. On the one hand, it involves distinctive factors in relation to women themselves, that is, the psychological effects brought about by physiological changes in women at different life stages (menstrual, pregnancy, lactation, and menopause) (6). Research has shown that postpartum depression (PPD) is one of the most common mental health problems in postpartum women and may lead to negative emotions, such as low mood, anxiety, and exhaustion (7). On the other hand, women have long been pressured by conflicting social roles and gender expectations within cultures (8). In daily life, women influenced by traditional cultural perceptions may face the risk of being deprived of educational opportunities during their upbringing and ongoing life (9). In marriage, they may have to face anxieties related to children, in-laws, and age (10). In the workplace, the presence of gender bias and discrimination places women at a disadvantage in employment, job competition, promotions, and salary increase (11). Evidence shows that men earn $1.29 for every $1 women earn in management positions (12), and women are less likely to advance in the workplace. Although the quest for gender equality has become a global initiative, the deeply entrenched influence of traditional cultural institutions still subtly hinders the prospects of fairness and justice for women in life and work, making it likely that they will continue to face “work-family” dual pressure for some time to come (13, 14).

Previous studies have focused on the causal factors [physical characteristics (6) and external factors (15)], influencing factors [natural (16), social (17), and developmental factors (18), and heterogeneous factors (role (19), regional differences (20), and gender differences (21)] affecting women’s mental health problems. In terms of regional differences, different from the conclusion that the mental health status of rural residents was better than that of urban residents in western developed countries (22), the mental health status of rural residents in China, especially women, was worse than that of urban residents (23–25). These urban–rural differences stem from China’s unique household registration system, the urban–rural dual system, unbalanced socio-economic development, and gender relations (26). Notably, although the causes and influencing factors of women’s mental health problems are diverse, they fundamentally arise from the process of women seeking livelihoods in their daily lives, and are related to the availability and sustainability of livelihood capital (capital that can determine an individual’s ability and strategy to cope with environmental changes). Yet, few studies systematically explore how livelihood capital-encompassing human, social, physical, and financial resources interact to differentially impact urban and rural women’s mental health. Furthermore, the mediating role of relative deprivation has not been fully explored in the mechanism linking livelihood capital and mental health (27). Additionally, a sense of relative deprivation in terms of access to livelihood capital (an individual’s sense of disadvantage in socioeconomic comparisons) may have a negative impact on mental health. Although previous studies have explored the relationship between livelihood capital and women’s mental health, the systematic role of livelihood capital has tended to be explored from a single perspective only, without a comprehensive analysis of multiple factors. Furthermore, the mediating role of relative deprivation has not been fully explored in the mechanism linking livelihood capital and mental health. Moreover, there is limited research on the mechanisms and geographical differences involved in terms of the effects of livelihood capital on women’s mental health. Likewise, when researching the impact of livelihood capital on mental health, the China Labor—force Dynamics Survey (CLDS) is highly advantageous (28). It comprehensively covers variables related to livelihood capital, mental health, and relative deprivation, and its large, representative sample (using a scientific sampling method across 29 provinces and municipalities as in the 2018 CLDS offers strong data foundation (29). Therefore, through using the 2018 China Labor-force Dynamics Survey (CLDS), combined with structural equation model, this study aimed to explore the relationships and pathways between livelihood capital elements and women’s mental health and the mediation effect of relative deprivation. The findings are expected to help better understand women’s mental health needs in periods of social change and promote more effective and equitable social integration to address such needs. This study advocates for tailored interventions that address both livelihood capital and psychosocial well-being. In doing so, we bridge the gap between structural and psychological perspectives on women’s mental health, offering a comprehensive lens for future research and practice.

## 2 Review

### 2.1 Measuring women’s mental health

The World Health Organization defines mental health as a state of well-being that enables people to cope with the stress of life, achieve their potential, work effectively, and express individual and social values (30). The measurement of mental health, which differed from physical health in terms of being a more relative and dynamic concept, depends on an individual’s emotional, cognitive, and behavioral adaptability to the environment (31). Mental health is commonly measured by self-report questionnaires because of their ease of implementation, simplicity of operation, low cost, and availability of large amounts of data in a short time (32). Such questionnaires include the Baker Depression Scale (BDI) (33), Generalized Anxiety Scale-7 (GAD-7) (34), and the Center for Epidemiologic Studies Depression Scale-20 (CES-D-20) (34). Among them, the CES-D-20 scale was not only time-sensitive and convenient for assessing depressive symptoms (35) but was also widely applicable to different populations worldwide because of its cross-cultural adaptability (36, 37).

Women’s mental health is an important branch of mental health. Based on the three-factor discrimination method used in relation to bisexuality and psychological problems (24, 25), this study considered that women’s mental health refers to a harmonious and integrated female mental state, comprising intellectual development, social interaction, and moral refinement. The primary indicators of women’s mental health included a positive spirit, strong will, sound personality, normal intelligence, principled morality, harmonious interpersonal relations, moderate social responsiveness, and effective psychological performance in line with age characteristics (26). Due to the interaction of complex factors such as women’s specific gender characteristics and gender socialization (5), women have a vulnerable position in mental health issues. Compared with men, women have a higher prevalence of mental disorders, including depression, anxiety, PTSD, etc (1). Therefore. The research on women’s mental health has attracted much attention from scholars. The developmental process of women’s mental health research has mainly comprised three important stages (embryonic, developmental, and mature stages). In the embryonic stage (1900s−1970), clinical psychology research focused more on emotional disorders and psychopathological problems arising from the physiological characteristics of women. Seeman et al. (38) found that periodic fluctuations in estrogen and progesterone levels enhanced the stress response, thus enhancing susceptibility to depression and anxiety. In the developmental stage (1980–2000), with the rise in gender difference research, scholars began to pay attention to the characteristics and disadvantages arising in relation to women’s mental health and explored ways to help women cope better with stress through gender-sensitive interventions (39, 40). Nolen et al. (41) found that gender differences significantly affected emotion regulation strategies and mental health. Compared to men, women’s more negative coping styles significantly increased the risk of depression. In the mature stage (2000–present), research on women’s mental health gradually developed from an early pathological perspective to a comprehensive viewpoint covering physiological, psychological, social, and cultural dimensions (42). Research suggests that a strong social network that acts as a buffer may help pregnant women cope with the stress associated with pregnancy and childbirth, thereby mitigating the negative effects of psychological distress (43).

### 2.2 Livelihood capital, relative deprivation, and women’s mental health

“Livelihood” is defined as the capabilities, assets (including physical and social resources) and activities required for a means of living. “Livelihood capitals” refer to the resource base of a community and of different categories of households (44). Livelihood capital largely determined an individual’s ability and strategies for coping with environmental changes (45, 46). For women, access to the sustainability of livelihood capital played vital roles in coping with social, economic, and family pressures. Livelihood capital was measured from five aspects: natural capital (natural resources, such as land, water, forests, and fisheries), physical capital (production equipment, housing, and infrastructure), human capital (labor, skills, knowledge, and health status), social capital (social relationships, networks, trust, and mutual aid mechanisms), and financial capital (financial assets, such as cash, savings, and credit) (47, 48). These five forms of capital affected individual mental health in different dimensions.

In terms of natural capital and from the perspective of livelihood dependence, having stable natural resources mean less livelihood uncertainty with accompanying anxiety and pressure (49), while exposure to the natural environment contributed to psychological recovery and cognitive function improvement. Research has found that exposure to various natural stimuli can consistently improve cognitive performance and promote positive emotions (50). Regarding physical capital, the discomforting experiences brought about by commuting demands and poor housing conditions (e.g., in terms of ventilation, lighting, and overcrowding) may increase anxiety (15, 51). A study conducted in Shanghai found that the mental health status of female commuters was significantly and negatively correlated with a longer work-residence distance (51). At the same time, human capital factors, such as education level and work skills, were key influences on mental health. Pearlin et al. (52) reported that higher education and career prestige reduced the negative impact of other stressors on mental health. Additionally, greater social capital fostered stress relief and improved life satisfaction, mainly through social interaction (53). Pattussi et al. (54) found that social cohesion in social interaction played an important role in promoting the mental health of female employees. Zhang et al. (55) found that neighborhood social interaction had a positive impact on the mental health of women, and the degree of impact was higher than that in men. Finally, the impact of financial capital on mental health was reflected in effective financial management in relation to income, insurance, and other factors (56). Kose et al. (57) found a positive correlation between family income and women’s mental health status in Turkey, and Turunen et al. (58) found that debt mediated the effect of income on mental health when income decreased. Another study found that different types of insurance had different effects on improving mental health, with greater levels of social pension insurance having proportionally improving effects on mental health, whereas greater levels of commercial pension insurance had proportionally negative effects on mental health (59). Furthermore, some scholars have found that the impact of livelihood capital on mental health may vary due to regional differences. In urban areas, women often faced greater professional competition while balancing family responsibilities, which exacerbated mental health issues (19, 60). In contrast, rural women’s livelihood capital depended more on natural resources and traditional agricultural economies, and their mental health problems were closely related to resource scarcity and livelihood vulnerability (61). Due to the lack of sufficient economic and social support networks in rural areas, females in these regions were more prone to anxiety and depression when facing livelihood risks (62). Based on the above—mentioned relationships between different forms of livelihood capital and mental health, it can be seen that livelihood capital is likely to have a direct impact on women’s mental health. Accordingly, we propose the following hypothesis:

*Hypothesis 1:* Livelihood capital direct impact women’s mental health.

There may be complex interactions among feelings of relative deprivation, livelihood capital, and women’s mental health. According to Walker and Smith (27), a sense of relative deprivation referred to an individual’s perception of their own inferiority compared to a reference object. The preconditions for an individual’s sense of relative deprivation included: “the individual does not have the thing,” “the person being compared with has the thing,” “the individual wishes to have the thing,” and “the individual considers this expectation reasonable.” The things being compared, such as personal income and social status, can vary considerably (27). There was a lot of evidence that relative deprivation adversely affected mental health (63, 64). Mental health of low-income individuals was significantly worse than high-income individuals. The income-related mental-health gap has widened over time (65). A sense of relative deprivation has a negative effect on women’s mental health because, when individuals feel there was inequity between themselves and others in relation to their economic, social, or cultural status, this often produces a sense of helplessness and loss of control, leading to depression, anxiety, and other psychological problems (64). Especially in a social environment with a wide gap between the rich and poor, the psychological pressure and sense of relative deprivation can increase significantly among females in more advanced societies (66). Notably, there was an interactive relationship between relative deprivation and access to livelihood capital. People with inadequate livelihood capital are prone to feelings of relative deprivation, which might affect their ability to access livelihood capital. For example, people lacking social capital may be trapped in a vicious cycle of relative deprivation due to social exclusion, which made it more difficult for them to access the necessary financial or emotional support through social support networks (67). Thus, the following hypothesis can be claimed:

*Hypothesis 2:* Relative deprivation plays a mediating role in the relationship between livelihood capital and women’s mental health.

### 2.3 Understanding mental health in China: urban–rural differences

Urban and rural areas differ in the mental health of women. Previous Western studies on the mental health of urban and rural residents indicated that urban residents are more likely to suffer from mental illness (including depression, anxiety disorders, mood disorders, etc.) compared with rural regions (22). Women in urban areas of China also have a high labor force participation rate, which may contribute to their depression (68, 69). With the improvement of urbanization level, the risk of abnormal mental states and mental illness of residents increases (70). A possible explanation for these rural–urban differences in the West was the negative impact of urban environmental factors on mental health. Due to the deterioration of urban physical factors (air pollution, population density, housing conditions, etc.) and social factors (stress, life events, social isolation, etc.) brought about by urbanization, urban residents were more likely to cause negative mental states (22). At the same time, Western developed countries had been in the process of counter-urbanization, high-income groups were more likely to live in suburbs or rural regions with better environments and security, and urban regions were more concentrated by low-income groups. And groups with better socioeconomic status were more likely to have good mental health (71). The interaction between the living environment and individual characteristics led to the differences between the urban and rural mental health of Western residents.

However, as a typical developing country, China differed substantially from Western developed countries in terms of economy, culture, system, and gender relations. This made the difference in mental health status between urban and rural residents in China special. The mental health status of urban residents in China was better than that of rural residents. Compared with urban residents, Chinese rural residents had a higher risk of suicide and mental health disorders (23, 24). The main reasons for this difference in mental health between urban and rural residents in China were the lasting impact of the dual urban–rural administrative system, *hukou* (household registration) policies, and unbalanced economic development. China’s long-standing *hukou* policies made the transition from rural to urban hukou rather difficult (72). Compared with rural regions, Chinese urban residents enjoyed more socioeconomic benefits, such as more medical resources, better education facilities, and a more convenient living environment (73); in contrast, rural residents suffered from lagging socio-economic development, low levels of education, fewer employment opportunities, inadequate access to health services, environmental hazards and economic hardships (24). The rapid urbanization in China has exacerbated this imbalance between urban and rural areas (74), further affecting residents’ mental health. In this context of regional economic imbalance, the special situation of urban and rural women deserves attention. Due to the continuous influence of patriarchal traditional culture on Chinese society, the gender norm of “son preference” has been shaped, putting women at a disadvantage in terms of social status, family power, division of labor, and access to educational resources for a long time. Moreover, the acceleration of urbanization and marketization has further emphasized efficiency, productivity, and profit-orientation, forcing women into a more vulnerable position (75). Rural Chinese women had lower levels of mental health than their urban counterparts. Studies have shown that the suicide rate for women in China was 25 percent higher than for men, and the suicide rate for young women in rural areas was higher than in urban areas (25). Long-term exposure to such discriminatory cultures by patriarchal, son preference and subordinate status of women will lead to psychological stress in the socialization process (26). The lower mental health level of rural women may be due to the deeper influence of this traditional patriarchal culture. Given the significant urban–rural differences in China in terms of economic development, social environment, and the mental health status of women. Thus, the following hypothesis is put forward:

*Hypothesis 3:* Livelihood capital of direct and indirect impact on women’s mental health has Urban–Rural differences.

**Figure 1:**
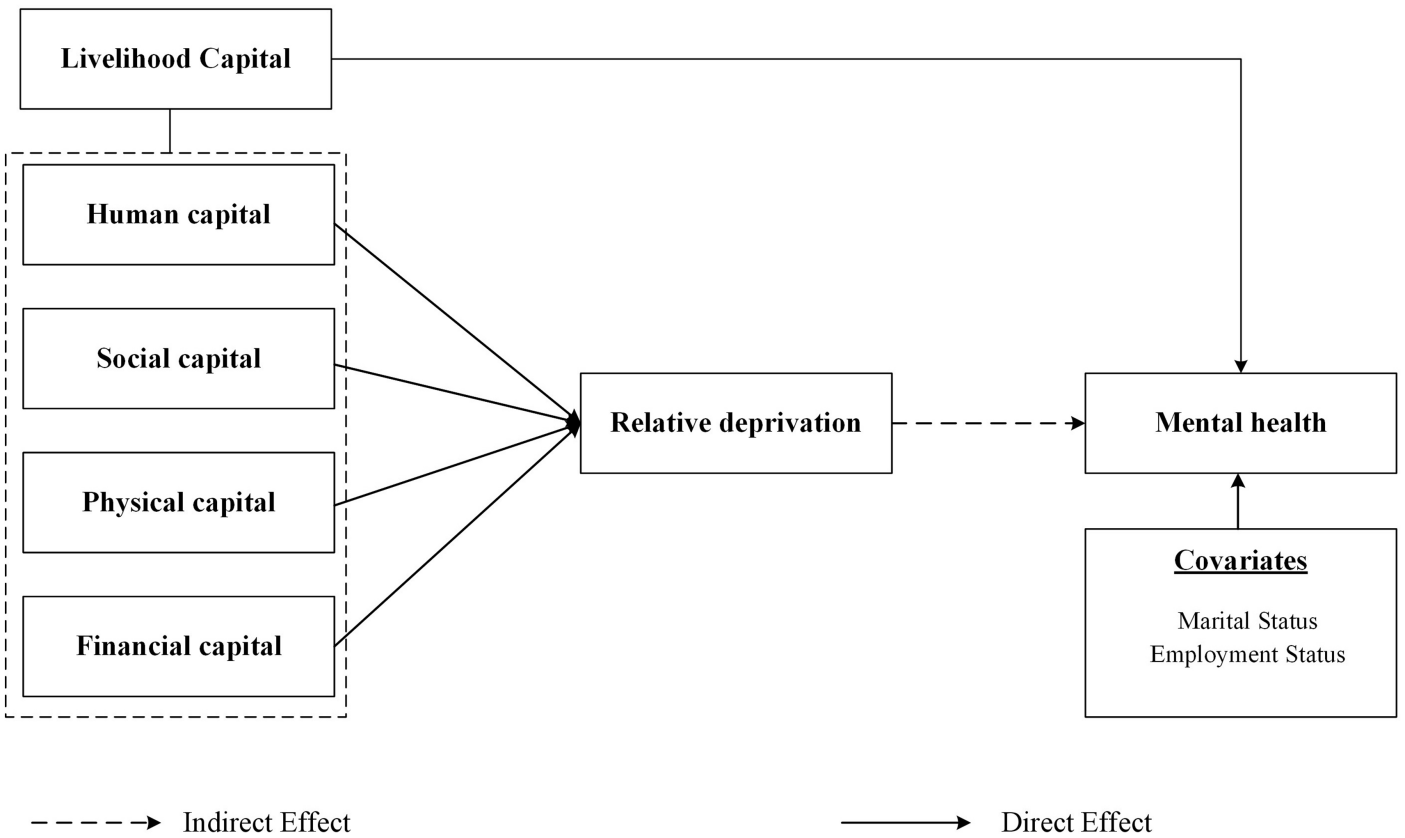
Conceptual framework.

In summary, the current academic research on women’s mental health has developed into a comprehensive perspective covering physiological, psychological, social, and cultural dimensions. However, the existing research mostly discusses the impact of livelihood capital on women’s mental health from a single perspective, lacking a systematic comprehensive analysis of livelihood capital from multiple perspectives. Meanwhile, livelihood capital is associated with relative deprivation. The pathway mechanism by which livelihood capital brings relative deprivation and thus affects women’s mental health also needs to be considered. Additionally, considering China’s urban–rural dual hukou policy, the social background of unbalanced economic development, and the special situation of women, urban and rural women may be affected differently by livelihood capital. Therefore, based on CLDS data, this study will explore the mechanism of livelihood capital on women’s mental health from four aspects: human capital, social capital, Physical capital, and financial capital. And the mediating role of relative deprivation and urban–rural differences was also considered. In the process, we can effectively bridge the gap between structural and psychological perspectives on women’s mental health. This not only provides a more comprehensive understanding of the issue but also offers a multifaceted lens for future research and practical applications. It enables researchers to explore more nuanced relationships between various factors and mental health, and practitioners to design more targeted and impactful interventions (Figure 1).

## 3 Data and methods

### 3.1 Data

This study used data from the 2018 China Labor-force Dynamics Survey (CLDS) which conducted by the Center for Social Survey of Sun Yat-sen University (CSS). The survey is a national and representative dataset targeted 16,537 individuals aged 15–64 years and used a multistage, stratified probability proportionate-to-labor force size sampling technique to ensure the representativeness of the samples. In addition, CLDS data protected experiment ethics and conceals private information. The CLDS achieved broad coverage across 29 provinces and municipalities. Specifically, it chose 110 sample cities with more than 25 participants as the study subjects and encompassed three key dimensions: individuals, families, and community. It comprehensively gathered information related to employment, demographics, and economic and social aspects. The uniqueness of the survey lies in its comprehensive data collection, extending from the labor force to the family and society generally, providing a multidimensional and multiple-level research foundation. In the context of this research topic, this study specifically focused on the sample data concerning women. After excluding samples with missing values that were not suitable for this study, we obtained 5,278 samples, which were representative. The scale and representativeness of these data provided a solid foundation for the study, offering detailed and reliable insights into the status of women in the labor market and in society.

The distribution of socio-demographic characteristics of the sample was summarized in Table 1. The mean age of the participants was 46.35 years, with most (84.8%) being married. With respect to employment status, employed individuals comprised a large proportion (91.5%). Education backgrounds vary among participants, with 43.6% having completed high school, 11.1% being college or above graduates, and 45.3 falling into “primary school and lower” category. Nearly one out of six respondents had a college’s degree or higher. Immigrants constituted a major proportion of the survey sample (86.1%), the majority of whom had rural household registrations (83.5%). A total of 58.6% of participants had a annal income of 35,000 yuan or less. In terms of class affiliation, 3.4% belonged to communist party member. Table 1 also presented the sociodemographic characteristics of two sub-samples (urban and rural women). The rural women had a higher mean age (46.59 years) and a higher proportion of married individuals (86.1%). Regarding education, the education level of the urban women (36.5% with college or higher) was higher than that of the rural women (6.0% with college or higher). With regard to employment status, rural women demonstrated a higher degree of participation in employment rate, as evidenced by the proportion being 91.5%. Moreover, in terms of individual annual income, the proportion of the rural women which earned 60,000 yuan or more (30.8%) was relatively lower than urban women (36%). Compared with the rural women, the urban women had higher incomes. Overall, the survey respondents’ characteristics indicated that identification of the factors affecting women’s mental health in different regions was comprehensive and aligned well with the features of the national female population. The study was representative, with notable urban–rural differences identified.

**Table 1:** Socio-demographic characteristics of participants.

	Female Residents (*N* = 5,278)	Urban Women Residents (*N* = 872)	Rural Women Residents (*N* = 4,406)
Age (years)			
Means	46.35	45.13	46.59
Education Attainment (%)			
Primary school or lower	45.3	10.8	52.2
High school	43.6	52.7	41.8
College or above	11.1	36.5	6.0
Marital status (%)			
Single	11.6	16.2	10.7
Married	84.8	78.2	86.1
Divorced/Widowed	3.6	5.6	3.2
Employment status (%)			
Employed	91.5	91.2	91.5
Unemployed	8.5	8.8	8.5
Hukou (%)			
Local Hukou	13.9	28.1	11.1
Non-local Hukou	86.1	71.9	88.9
Personal annual income (yuan; %)			
<12,000	34.4	18	37.6
12,001–36,000	24.2	28.9	23.3
36,000–60,000	9.7	17.1	8.3
60,001–120,000	4.6	11.3	3.2
>120,001	27.1	24.7	27.6
Political Affiliation (%)			
Communist Party member	3.4	12.4	1.6
Non-Communist Party member	96.6	87.6	98.4

“Local Hukou” represents household registration in this city.

### 3.2 Variables

#### 3.2.1 Mental health

Currently, evaluating mental health was not guided by a single, standardized approach. Commonly used mental health measures include the General Health Questionnaire (GHQ-12), Symptom Checklist, CES-D-20, Anxiety Self-Rating Scale, and Cornell Medical Index (CMI) Self-Rating Scale. This study used the CES-D-20 and the 2018 CLDS to assess women’s mental health, which has been used in previous studies to investigate factors affecting the severity of depression in young people (74). The CES-D-20 contains 20 items used to assess depression symptoms and is scored on a four-point reverse scale (1 = almost always or 5–7 days per week; 2 = often or 3–4 days per week; 3 = rarely or 1–2 days per week; and 4 = almost never or < 1 day per week) (76). The total depression score ranges from 20 to 80, with higher scores indicating more severe depression and poorer mental health. Moreover, the criteria for depression are as follows: no depression symptoms (depression score ≤ 35), possible depression symptoms (depression score ranging from 36 to 39), and existing depression symptoms (depression score ≥ 35). The original CES-D-20 questionnaire was divided into four factors: depression (items 1, 3, 6, 9, 10, 14, 17, and 18), positive affect (items 4, 8, 12, and 16), somatic symptoms/activity inhibition (items 2, 5, 7, 11, 13, and 20), and interpersonal problems (items 15 and 19). Subsequent data processing standardized the questionnaire such that all items were inverted. The Cronbach’s alpha indicated high internal consistency among the research questionnaires (0.952).

#### 3.2.2 Independent variables

This study drew on the livelihood capital theorizing of the United Kingdom Department for International Development (DFID) to categorize the variables (48). The independent variables selected from the 2018 CLDS questionnaire were divided into four categories: human, social, physical, and financial capitals. The study choice indicators via previous studies. First, Liu (77), and Wang (78) used education, health and age to measure human capital. Moreover, according to previous studies (79–81), the study utilized neighbor trust, neighbor familiarity, social network and social trust to measure human capital. Additionally, Ren (82) measured physical capital by housing facilities, whereas Leah et al. (83) examined physical capital by housing conditions, such as ventilation, daylighting, air quality, and overcrowding. Last but not least, prior studies (84, 85) used income and insurance to gauge financial capital.

Human capital was assessed by educational level, self-rated health and age. This study assessed human capital by 3 dimensions and asked participants 3 questions: (1) “What is your educational level?” (educational level) (77) Responses were categorized as 1 = primary school or lower, 2 = high school, 3 = college or above; (2) “How would you rate your overall health?” (self-rated health) (77) Responses were categorized as 5 = very healthy, 4 = healthy, 3 = fair, 2 = unhealthy, 1 = very unhealthy; (3) “How old are you?” (age) (78).

Social capital mainly consisted of three parts (network, trust, and reciprocity), indicating the numerous social resources upon which individuals achieved their livelihood objectives (48), which was measured in this study from four aspects: neighbor trust (79), neighbor familiarity (80), social network (81), and social trust (79). Regarding the neighbor trust, the questionnaire includes “Do you trust the neighbors, neighborhood, and other residents in your community (village)” (1 = very distrust, 2 = not very trust, 3 = general, 4 = relatively trust, 5 = very trust). In terms of neighbor familiarity, the following question was asked in the questionnaire: “How well do you know your neighbors, neighborhood, and other residents in your community (village)?” (1 = very unfamiliar, 2 = not very unfamiliar, 3 = general, 4 = relatively familiar, 5 = very familiar). Regarding the social network, the questionnaire includes “How often does mutual help occur between you and your neighbors and other residents of this community?” with options on an ascending scale from 1 = never to 5 = always. After omitting missing values, the study divided social trust from the perspective of specific objects into five categories. They were trust in, respectively, family members, neighbors, schoolmates, fellow villagers, colleagues, rated by level of trust from low to high (5 = completely untrustworthy, 25 = completely trustworthy). The social trust demonstrated a high internal consistency (Cronbach’s alpha = 0.823).

Physical capital was reflected in the infrastructure and material resources to sustain individuals’ livelihoods and enhance their labor productivity (48). In this study, the assessment of physical capital by four variables. (1) “room-base ventilation” (83): Natural ventilation inside the house was measured in this survey, with 10-levels answers (lower values, poor ventilation); (2) “indoor daylight quality” (83): the quality of architectural daylight inside the house (1–10 points, higher values, better quality); (3) “overcrowding” (83): overcrowding condition inside the house (1–10 points, lower values, more overcrowding); (4) “indoor air quality” (83): the quality of air inside the house (1–10 points, higher values, better quality); (5) “housing facilities” (82): the number of facilities in the house.

Financial capital referred to different financial resources, including sources of finance and credit opportunities for production and consumption (48). Financial capital was measured by three indicators: personal annual income (84), housing provident funds (no housing provident funds = 0; owns housing provident funds = 1) (85), and industrial accident compensation insurance (no industrial accident compensation insurance =0; owns industrial accident compensation insurance = 1) (85).

#### 3.2.3 Mediator variable

This study examined potential pathways through which livelihood capital could affect the mental health of women, with relative deprivation selected as a mediator variable and measured using the MacArthur Scale (86). The participants were asked to describe their position in a self-defined society on an upright ladder with a hierarchy comprising 10 levels (1 = bottom; 10 = top bottom). Relative deprivation was measured using the following question: “Where do you currently see yourself in the hierarchy?,” “Where do you see yourself in the hierarchy 5 years ago?,” “Where do you see yourself in the hierarchy in 5 years?” and “Where do you see your household in the hierarchy currently?” Higher total scores indicated lower perceived relative deprivation.

#### 3.2.4 Covariates

Two demographic characteristics constituted the covariates. Marital status was coded as a factor variable categorized into single, married, divorced/widowed, respectively. Employment status was categorized into currently employed (including full-time, part-time or casual work) or unemployed (including studying, home duties, and retired). The detailed metrics for all variables were listed in Table 2.

**Table 2:** Detailed metrics of variables.

Variable Name	Dimension	Indicator	Definition
Human Capital	HC1	Educational Level (77)	The educational level. 1 = primary school or lower, 2 = high school, 3 = college or above
	HC2	Health (77)	The self-rated health. 1 = very unhealthy, 2 = unhealthy, 3 = fair, 4 = healthy, 5 = very healthy
	HC3	Age (78)	The age (year).
Social Capital	SC1	Neighbor Trust (79)	Trust in neighbors. (1 = very distrust, 2 = not very trust, 3 = general, 4 = relatively trust, 5 = very trust)
	SC2	Neighbor Familiarity (80)	Familiar with neighbors. (1 = very unfamiliar, 2 = not very unfamiliar, 3 = general, 4 = relatively familiar, 5 = very familiar)
	SC3	Social Network (81)	“How often does mutual help occur between you and your neighbors and other residents of this community?” with options on an ascending scale from 1 = never to 5 = always.
	SC4	Social Trust (79)	Social trust was divided into family members, neighbors, schoolmates, fellow villagers, colleagues, rated by level of trust from low to high (from 5 = completely untrustworthy to 25 = completely trustworthy).
Physical Capital	PC1	Room-based Ventilation (83)	Natural ventilation inside the house (1–10 points, lower values, poor ventilation)
	PC2	Indoor Daylight Quality (83)	Architectural daylight inside the house (1–10 points, higher values, better quality)
	PC3	Housing Facilities (82)	Number of facilities in the house
	PC4	Overcrowding (83)	Overcrowding inside the house (1–10 points, lower values, more overcrowding)
	PC5	Indoor Air Quality (83)	Air quality inside the house (1–10 points, higher values, better quality)
Financial Capital	FC1	Housing Provident Fund (85)	Purchase housing provident fund; 1 = yes, 0 = no
	FC2	Personal Annual Income (84)	Total annual income in 2017
	FC3	Industrial Accident Compensation Insurance (85)	Purchase industrial injury insurance; 1 = yes, 0 = no
Mental Health	Mental Health	Depression	The occurrence of 20 conditions in the past week: “I am troubled by some trivial stuff,” “I have not appetite to eat,” “Even if my family and friends help me, I still cannot get rid of my depression,” “I feel I’m worse than most people,” “I’m depressed,” “I cannot concentrate when I do things,” “I feel exhausted with everything,” “I feel hopeless,” “I think my life is a failure,” “My sleep quality is bad,”“I’m scared,” “I feel unhappy,” “I do not talk as much as usual,” “I feel lonely,” “I feel my life is boring,” “I feel people are not very kind to me,” “I used to cry,” “I feel apprehensive,” “I do not feel liked by people,” and “I do not think I can go about my daily work.” Reverse scoring depression scores into mental health scores(20–80 points)
Relative Deprivation	Relative Deprivation	Relative Deprivation	The position in a self-defined society on a picture of an upright ladder with 10 rungs—with a score of “1” representing the bottom rung and a score of “10” representing the top rung.

### 3.3 Statistical analysis

This study used structural equation modeling (SEM) to examine the relationship between livelihood capital (including human, social, physical, and financial capital), relative deprivation, and women’s mental health. SEM can be thought of as a mixture of regression and factor analysis to analyze both the factors affecting women’s mental health and the influencing pathways of women’s mental health (87). For example, Feng et al. (88) used structural equation modeling (SEM) to assess the relationship between urbanization, four mediators, and depressive symptom severity. SEM can assess the total, direct, and indirect effects of one variable (e.g., human, social, physical, and financial capital) on another variable (e.g., women’s mental health) and was used in our study to explore the potential mechanisms between livelihood capital and women’s mental health (89). In the SEM models, human, social, physical, and financial capital were set as exogenous variables. Relative deprivation and women’s mental health were set as endogenous variables. To construct models for SEM analysis, causal diagrams hypothesized *a priori* from the literature review was initially formed to depict the mediating effects of livelihood capital and relative deprivation on women’s mental health. The associations between the initial model variables and women’s mental health were examined using linear regression.

This study used SPSS Amos 26 software for SEM, STATA version 18, and SPSS version 27 software for basic pre-analysis data cleaning. This study considered existing research to examine the fit parameters for the SEM, which tested the proposed models using the following model fit parameter criteria: the chi-square to degrees of freedom ratio (CMIN/DF) ≤5; root mean square error of approximation (RMSEA) ≤0.08; incremental fit index (IFI) ≥0.90; Tucker-Lewis index (TLI) ≥0.90; and comparative fit index (CFI) ≥0.90. Bootstrapping was used to examine the mediating effect (5,000 resamples) in which the effect was deemed significant with a 95% confidence interval (CI) not including 0 (90).

## 4 Results

### 4.1 Structural equation analysis of total sample

The adequacy of fit was evaluated using confirmatory factor analysis (CFA). The model included four latent variables: human, social, physical, and financial capital. The results demonstrated a good model data fit, with chi-square = 14.413 (*p* < 0.01), CFI = 0.938, IFI = 0.938, TLI = 0.922, and RMSEA = 0.036 (Table 3). All factor loadings of the latent variables were significant at the *p* < 0.001 level, among which the factor loadings for human capital (−0.521–0.897), social capital (0.402–0.833), physical capital (0.283–928) and financial capital (0.397–0.834) were close to 0.36. The composite reliability (CR) values of the latent variables were 0.63, 0.78, 0.85, and 0.73, respectively, close to 0.7, while the average variance extracted (AVE) values were 0.40, 0.49, 0.56, and 0.49, respectively, all close to 0.5 (Table 4). The correlation coefficients of the paths were all less than the square root of the AVE (Table 5). The fit parameters indicated acceptable convergent validity of the model, as outlined in Section 3.3. Furthermore, multi-group analyses indicated that the model was invariant among urban and rural women at the configural, metric, and scalar levels.

**Table 3:** Goodness-of-fit indices for structural model.

	*χ*^2^/df	RMSEA	CFI	IFI	TLI
Model values	14.413	0.036	0.938	0.938	0.922
Recommended values	<5	<0.8	>0.9	>0.9	>0.9

**Table 4:** Convergent validity of measurement model.

	Convergent validity		
Model Pathways	Estimate	AVE	CR
HC1 ← Human Capital	0.897***	0.3943	0.6249
HC2 ← Human Capital	0.32***		
HC3 ← Human Capital	−0.521***		
SC1 ← Social Capital	0.833***	0.4878	0.7763
SC2 ← Social Capital	0.734***		
SC3 ← Social Capital	0.73***		
SC4 ← Social Capital	0.402***		
PC1 ← Physical Capital	0.928***	0.558	0.8515
PC2 ← Physical Capital	0.821***		
PC3 ← Physical Capital	0.283***		
PC4 ← Physical Capital	0.721***		
PC5 ← Physical Capital	0.809***		
FC1 ← Financial Capital	0.782***	0.4858	0.7237
FC2 ← Financial Capital	0.397***		
FC3 ← Financial Capital	0.834***		

∗*p* < 0.1; ∗∗*p* < 0.05; ∗∗∗*p* < 0.01. HC1, Educational Level; HC2, Health; HC3, Age; SC1, Neighbor Trust; SC2, Neighbor Familiarity; SC3, Social Network; SC4, Social Trust; PC1, Room-based Ventilation; PC2, Indoor Daylight Quality; PC3, Housing Facilities; PC4, Overcrowding; PC5, Indoor Air Quality; FC1, Housing Provident Fund; FC2, Personal Annual Income; FC3, Industrial Accident Compensation Insurance.

**Table 5:** Discriminant validity of measurement model.

Constructs	Human Capital	Social Capital	Physical Capital	Financial Capital
Human Capital	0.3943			
Social Capital	−0.277***	0.4787		
Physical Capital	0.166***	0.021	0.558	
Financial Capital	0.578***	−0.181***	0.112***	0.4858
the Square Root of the AVE	0.63	0.69	0.75	0.70

∗*p* < 0.1; ∗∗*p* < 0.05; ∗∗∗*p* < 0.01.

**Figure 2:**
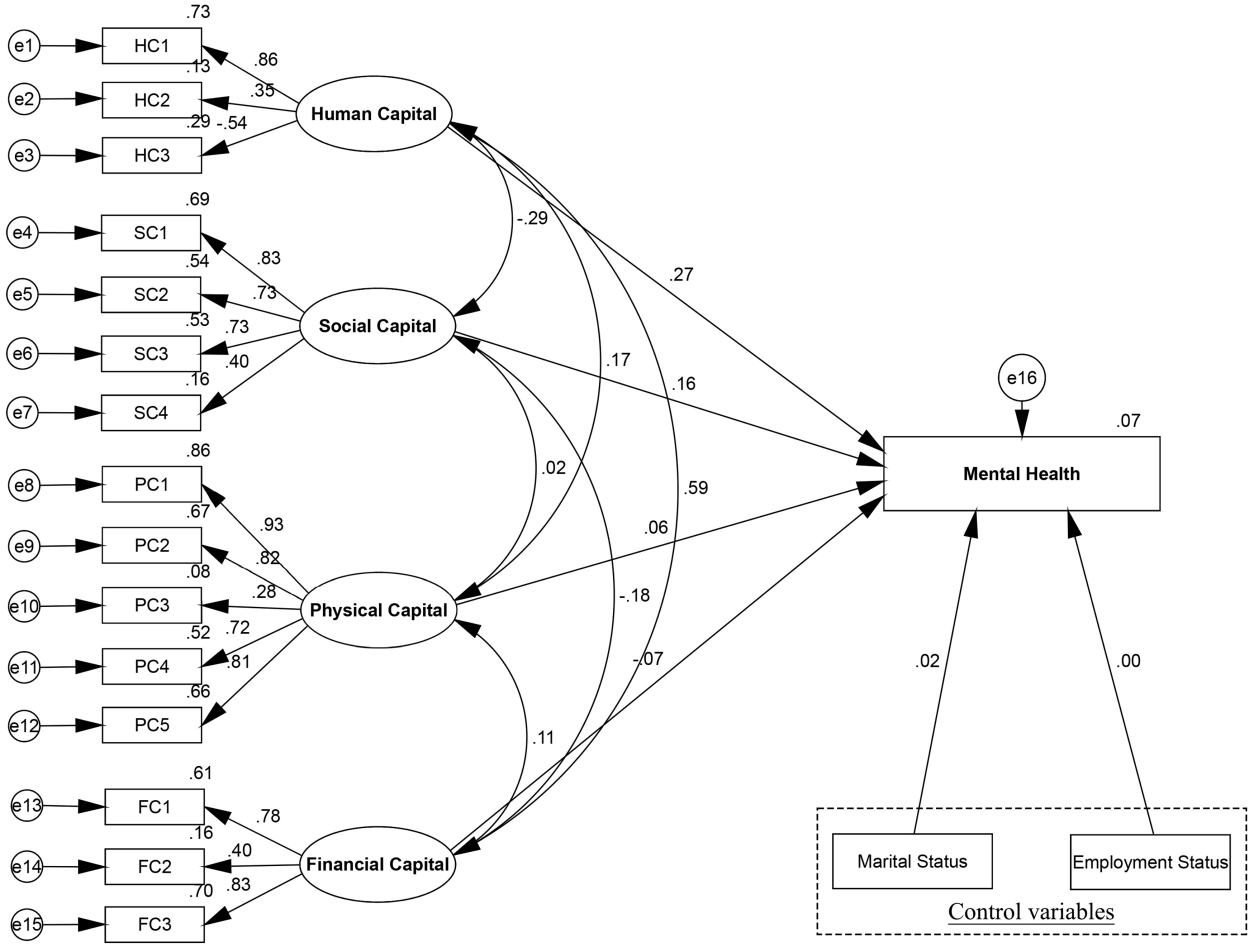
Structural equation modeling results of total women’s mental health.

This study used SEM to determine the relationship between livelihood capital and women’s mental health according to the fit indicators of the total model (Figure 2). Table 6 illustrated the results of the structural equation, which indicated that various elements of livelihood capital significantly affected women’s mental health, which supported to H1. The total model estimated the association between livelihood capital and women’s mental health while adjusting for covariates. The results showed that human, social, physical, and financial capital had positive and significant effects on women’s mental health (*p* < 0.01), with standard coefficients of 0.270, 0.160, 0.056, and −0.075, respectively. These empirical results supported the H1: Livelihood capital direct impact women’s mental health. Specifically, this study examined that a one standard deviation increased in human capital increased women’s mental health by 0.269, and human capital increased women’s mental health. Social capital exerted a significantly positive effect on women’s mental health, indicating that lower social capital contributed to a higher probability of depression. Moreover, increasing physical capital, including greater amounts of housing facilities and improved conditions, had a positive effect on women’s mental health. In terms of financial capital, this study provided evidence that financial capital had a direct negative effect on reported women’s mental health, indicating that changes in income and social security altered women’s mental health.

**Table 6:** The association between human capital, social capital, physical capital, financial capital and women’s mental health.

Model Pathways	Total Residents	Rural Residents	Urban Residents
	Coef. (SE)	Coef. (SE)	Coef. (SE)
Mental Health ← Human Capital	0.270*** (0.269)	0.267*** (0.330)	0.190** (0.672)
Mental Health ← Social Capital	0.160*** (0.224)	0.177*** (0.258)	0.111** (0.453)
Mental Health ← Physical Capital	0.056*** (0.091)	0.056*** (0.099)	0.038 (0.214)
Mental Health ← Financial Capital	−0.075*** (1.099)	−0.043 (1.723)	−0.119* (1.519)
Covariates			
Mental Health ← Marital Status	0.016 (0.337)	−0.018 (0.393)	0.063 (0.606)
Mental Health ← Current Working Status	−0.003 (0.460)	−0.005 (0.515)	0.013 (0.972)

Coef., coefficient; SE, standard error. ∗*p* < 0.1; ∗∗*p* < 0.05; ∗∗∗*p* < 0.01.

The model was adjusted for all covariates shown in this table. All the coefficients are standardized regression coefficients.

### 4.2 Mediating effect of relative deprivation

Given that H1, which posited that certain elements of livelihood capital would significantly affect women’s mental health levels, was supported, the study further explored H2. Specifically, the study examined whether relative deprivation had a significant mediating effect on the relationship between livelihood capital factors and women’s mental health. This involved assessing whether varying degrees of social deprivation altered the extent to which livelihood capital factors affected women’s mental health. This study used bootstrapping with 5,000 resamples and a 95% CI to test the significance of direct, indirect, and total effects in the models.

**Figure 3:**
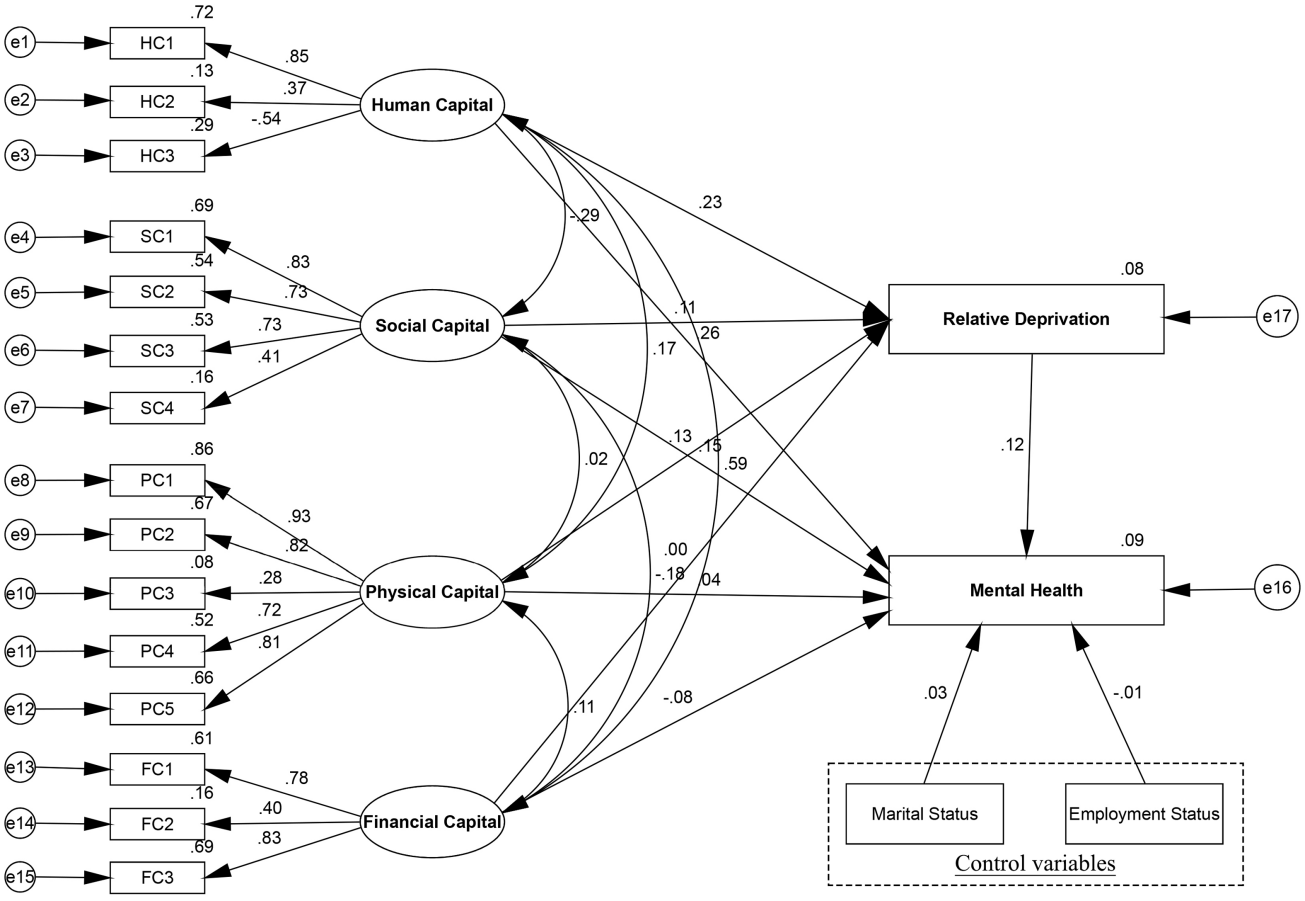
Mediation model of the association between livelihood capital and total women’s mental health through relative deprivation.

From the SEM results (Figure 3; Table 7), the study found that relative deprivation had a significant mediating association in the total model on human, social, and physical capital in terms of effects on women’s mental health, which supported H2. First, greater human capital was related to lower levels of relative deprivation, which in turn was associated with higher women’s mental health. This indirect effect was significant with a standardized coefficient of 0.028 (95% bootstrapping CI [0.021, 0.037]), accounting for 9.79% of the total effect (coef. = 0.286, 95% bootstrapping CI [0.009, 0.019]), while 90.21% of the impact of human capital directly affected women’s mental health (coef. = 0.258, 95% bootstrapping CI [0.200, 0.037]). Additionally, the results revealed that both the direct (coef. = 0.150, 95% bootstrapping CI [0.115, 0.182]) and mediated effects (coef. = 0.013, 95% bootstrapping CI [0.009, 0.019]) of social capital on women’s mental health were significant. Among the total effects (coef. = 0.163, 95% bootstrapping CI [0.128, 0.196]), the direct effect accounted for 92.02% of the total effect and the indirect effect accounted for 7.98%. These findings indicated that relative deprivation partially mediated the relationship between social capital and women’s mental health. Furthermore, as can be observed from relative deprivation scores, physical capital had an indirect effect (coef. = 0.016, 95% bootstrapping CI [0.011, 0.022]) on women’s mental health. The standard direct effects path of physical capital with women’s mental health was 0.038 (95% bootstrapping CI [0.009, 0.069]), accounting for 70.37% of the total effect (coef. =0.054, 95% bootstrapping CI [0.024, 0.085]). In this vein, relative deprivation showed a partially mediating effect on the relationship between physical capital and women’s mental health. However, the study indicated no indirect effect (95% bootstrapping CI [−0.006, 0.005]) of financial capital on reported women’s mental health, and the mediating role of relative deprivation was not substantiated. Comparatively, the proportion of indirect effect in the total effect of physical capital (29.63%) was higher than that of human capital (9.79%) and social capital (7.98%) on women’s mental health.

**Table 7:** The direct effect, indirect effect and total effect of livelihood on mental health.

Model Pathways	Coef.	Product of coefficients		Bias-Corrected 95% CI	
		SE	Z	Lower	Upper
Total samples					
**Total Effect**					
Mental Health ← Human Capital	0.286	0.030	9.53	0.227	0.345
Mental Health ← Social Capital	0.163	0.017	9.59	0.128	0.196
Mental Health ← Physical Capital	0.054	0.015	3.60	0.024	0.085
Mental Health ← Financial Capital	−0.083	0.022	−3.77	−0.127	−0.041
**Direct Effect**					
Mental Health ← Human Capital	0.258	0.030	8.60	0.200	0.317
Mental Health ← Social Capital	0.150	0.017	8.82	0.115	0.182
Mental Health ← Physical Capital	0.038	0.015	2.53	0.009	0.069
Mental Health ← Financial Capital	−0.082	0.022	−3.73	−0.126	−0.041
**Indirect Effect**					
Mental Health ← Human Capital	0.028	0.004	7.00	0.021	0.037
Mental Health ← Social Capital	0.013	0.002	6.50	0.009	0.019
Mental Health ← Physical Capital	0.016	0.003	5.33	0.011	0.022
Mental Health ← Financial Capital	0.000	0.003	0.00	−0.006	0.005
Rural samples					
**Total Effect**					
Mental Health ← Human Capital	0.273	0.027	10.11	0.220	0.328
Mental Health ← Social Capital	0.178	0.019	9.37	0.141	0.215
Mental Health ← Physical Capital	0.055	0.017	3.24	0.023	0.088
Mental Health ← Financial Capital	−0.045	0.020	−2.25	−0.089	−0.008
Direct Effect					
Mental Health ← Human Capital	0.247	0.027	9.15	0.194	0.301
Mental Health ← Social Capital	0.163	0.019	8.58	0.127	0.200
Mental Health ← Physical Capital	0.038	0.017	2.24	0.005	0.070
Mental Health ← Financial Capital	−0.045	0.020	−2.25	−0.089	−0.008
Indirect Effect					
Mental Health ← Human Capital	0.027	0.004	6.75	0.019	0.035
Mental Health ← Social Capital	0.015	0.003	5.00	0.010	0.021
Mental Health ← Physical Capital	0.018	0.003	6.00	0.012	0.025
Mental Health ← Financial Capital	−0.001	0.003	−0.33	−0.005	0.005
Urban samples					
**Total Effect**					
Mental Health ← Human Capital	0.204	0.076	2.68	0.066	0.363
Mental Health ← Social Capital	0.112	0.043	2.60	0.031	0.198
Mental Health ← Physical Capital	0.036	0.034	1.06	−0.030	0.102
Mental Health ← Financial Capital	−0.128	0.063	−2.03	−0.262	−0.015
**Direct Effect**					
Mental Health ← Human Capital	0.197	0.076	2.59	0.054	0.357
Mental Health ← Social Capital	0.109	0.043	2.53	0.024	0.195
Mental Health ← Physical Capital	0.033	0.034	0.97	−0.034	0.099
Mental Health ← Financial Capital	−0.127	0.063	−2.02	−0.262	−0.014
**Indirect Effect**					
Mental Health ← Human Capital	0.007	0.011	0.64	−0.012	0.031
Mental Health ← Social Capital	0.004	0.005	0.80	−0.005	0.017
Mental Health ← Physical Capital	0.003	0.005	0.60	−0.005	0.014
Mental Health ← Financial Capital	−0.001	0.003	−0.33	−0.014	0.002

5,000 bootstrap samples. Coef., coefficient; SE, standard error. The model was adjusted for all covariates shown in this table. All the coefficients are standardized regression coefficients.

### 4.3 Urban–rural differences

**Figure 4:**
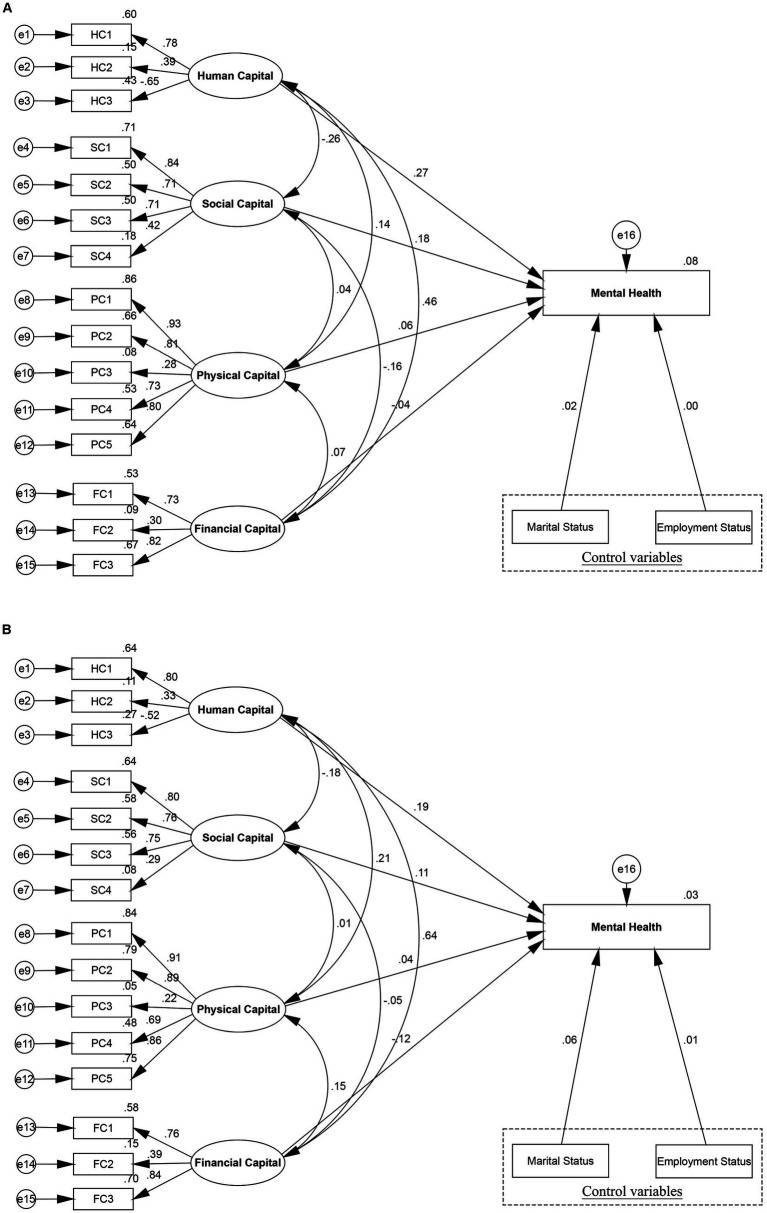
Structural equation modeling results of urban–rural differences. **(A)** Rural women’s mental health, **(B)** Urban women’s mental health.

T-test illustrated the significant difference of women’s mental health among urban and rural group, which were found to be statistically significant (*p* < 0.01). Figure 4 has identified the different effects of human, social, physical, and financial capital on women’s mental health among urban and rural women, which supported H3. Figure 4 and Table 6 revealed significant divergences across urban and rural women residents in the above structural paths. Specifically, human capital had a greater role in promoting rural women’s mental health (coef. = 0.267, *p* < 0.01) than urban women’s mental health (coef. = 0.190, *p* < 0.05). For each increase in the level of human capital, rural women’s mental health increased 0.077 grades more than urban women, which is significant at the level of 1%. Moreover, significant divergences were seen in the path coefficients of social capital to mental health between urban and rural subgroups (coef. = 0.111, *p* < 0.05, coef. = 0.177, *p* < 0.01, respectively). On the path connecting social capital to women’s mental health, the rural subgroup was more affected than the urban subgroup. Additionally, physical capital had a significant effect on women’s mental health of the rural subgroup (coef. = 0.056, *p* < 0.01), but it had no obvious effect on women’s mental health of the urban subgroup (coef. = 0.038, *p* > 0.1). However, compared to rural women (coef. = −0.043, *p* > 0.1), financial capital had a significant effect on women’s mental health in the urban subgroup (coef. = −0.119, *p* < 0.1). These findings reflected livelihood capital had significant differences between urban and rural women’s mental health in the different social and economic backgrounds.

**Figure 5:**
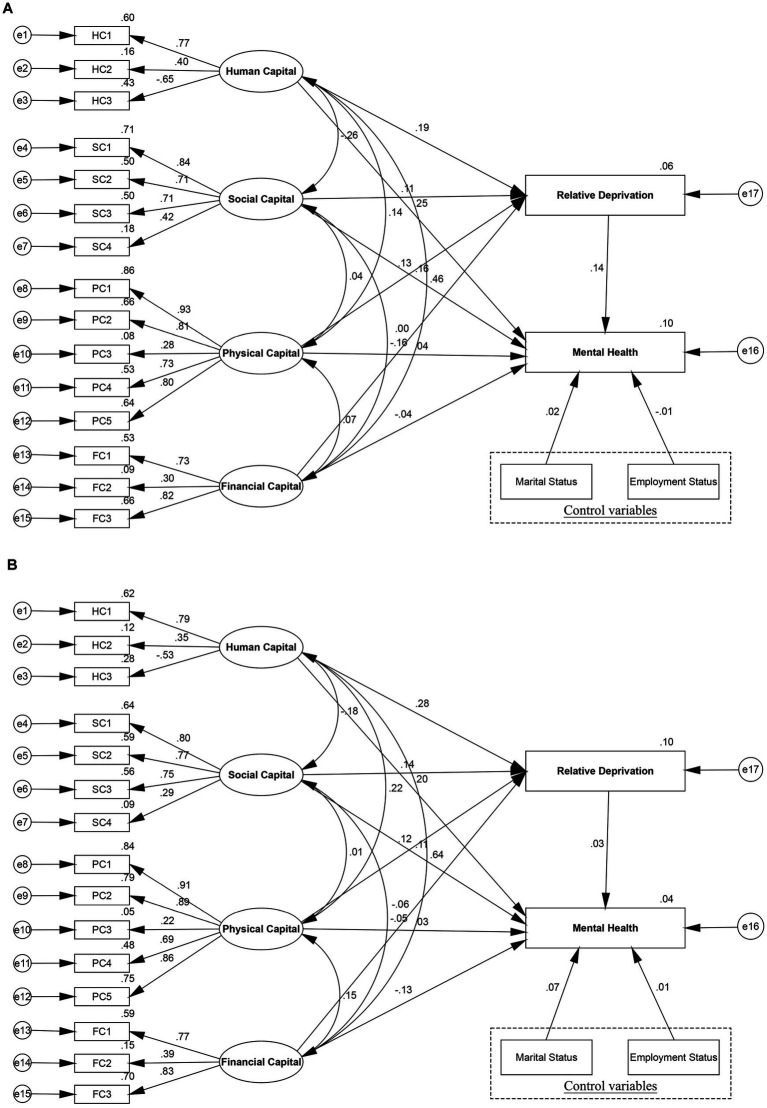
Mediation modeling results of urban–rural differences. **(A)** Rural women, **(B)** Urban women.

Based on the total model, the study compared the mediating effects for urban and rural subgroups to verify whether the relationship between livelihood capital and mental health differed, thereby further testing H3. The SEM of the rural subgroup (Figure 5) presented that relative deprivation had a mediating effect on the paths through which human, social, and physical capital influenced rural women’s mental health. However, in the SEM model for the urban sample (Figure 5), the CIs for the mediating effects of human, social, physical, and financial capital concluded 0, indicating that relative deprivation did not mediate the relationship between livelihood capital and mental health for the urban women. Thus, relative deprivation acted as a mediator for the rural women only (Table 7). Especially, the estimated direct effect (coef. = 0.247, 95% bootstrapping CI [0.194, 0.301]) of human capital on rural women’s mental health accounted for 90.48% of the total effect (coef. = 0.273, 95% bootstrapping CI [0.220, 0.328]), and 9.52% by the indirect path mediated by relative deprivation (coef. = 0.027, 95% bootstrapping CI [0.019, 0.035]). The relationship between social capital and women’s mental health was mediated by relative deprivation with indirect effect coefficients of 0.015 (95% bootstrapping CI [0.010, 0.021]), accounting for 8.43% of the total effect (coef. = 0.178, 95% bootstrapping CI [0.141, 0.215]). This result revealed the role of social capital in influencing women’s mental health, partly by reducing relative deprivation in rural women, thus improving mental health. What’s more, in the rural subgroup, significant associations were found for physical capital, relative deprivation, and women’s mental health. Relative deprivation mediated the greatest proportion in the model mediating physical capital and women’s mental health, with the indirect path (coef. = 0.018, 95% bootstrapping CI [0.012, 0.025]) accounting for 32.73% of the total path (coef. = 0.055, 95% bootstrapping CI [0.023, 0.088]). Finally, although financial capital had a direct effect on the rural subgroup (coef. = −0.045, 95% bootstrapping CI [−0.089, −0.008]), the indirect effect (95% bootstrapping CI [−0.005, 0.005]) remained not significant, indicating that financial capital was not mediated by relative deprivation.

## 5 Discussion

With rapid urbanization in China, it was a critical task to improve the quality of life of residents in various aspects. Increasing attention to mental health has highlighted the different requirements of various groups, particularly among women. Livelihood capital, a measure of the resources available to individuals or households for survival and development, has a significant impact on women’s mental health. In this context, this study focused on urban and rural women in China to explore the interactions between human capital, physical capital, social capital, financial capital, and relative deprivation with women’s mental health within the framework of livelihood capital. It also sought to elucidate the mediating mechanism of relative deprivation in the relationship between livelihood capital and women’s mental health, as well as between urban and rural women, to investigate differences in the mechanisms at play in various locations.

### 5.1 Livelihood capital and women’s mental health

The results investigated that livelihood capital had a significant positive effect on women’s mental health, confirming H1. In particular, enhanced human capital effectively improved mental health in women, which was consistent with previous findings (91). Human capital was significantly related to women’s mental health. The study focused on the finding that education, age, and health, as measures of human capital, considerably affected women’s mental health. Human capital reflected, to a certain extent, the skill levels, labor intensity, and status of women in the workplace (48). Higher education was associated with greater happiness and a reduction in psychological distress (92). First of all, education can cultivate long-term thinking skills and improve job skills. Women with high education level are more likely to engage in high-paying jobs, thus achieving higher happiness (93). Secondly, women with higher education are more likely to have more social interactions to reduce loneliness and get support, which has a positive impact on their mental health (94). Moreover, women’s health problems will also significantly affect their work performance and career development in the workplace, thus causing physical and mental burden on women (94). Aging can lead to various physical, functional, and social losses, increasing stress and negatively affecting women’s mental health and quality of life (95). Related to social capital, research found that social cohesion could improve women’s mental health, and neighborhood interaction had a positive correlation with women’s mental health (54). Noaparast (96) also reported that social capital played an important role in women’s mental health. Our findings were consistent with those of previous studies. This might be attributable to the social support that women receive during their social interactions. Women were likely to be affected by social support in neighborhood interactions, and greater trust in the community was likely to foster greater contentment (97). Social capital involved feelings of security and self-esteem, with improvements in these elements likely leading to improved women’s mental health (98).

In terms of physical capital, the study illustrated that physical capital related to housing conditions positively correlated with women’s mental health, which consistent with prior research (99). Housing overcrowding, insufficient lighting and ventilation, poor air quality, and inadequate housing facilities resulted in poorer women’s mental health because of higher rates of infectious disease transmission among those living in poor housing conditions (100). Housing overcrowding also reduced the chances of escape during disasters, and personal safety concerns were one of the causes of residents’ anxiety (101). Contrary to previous studies (102), this study found that financial capital negatively influenced women’s mental health. This discrepancy might be due to the fact that our sample did not differentiate between employed and unemployed women, whereas studies by researchers such as Kaori Honjo (103) focused on working women and homemakers, capturing a narrower range of female identities. This negative effect may be attributed to the non-linear relationship between wealth accumulation and mental health (104). Although the mental health problems of low-income groups are more prominent, high income may be accompanied by higher work pressure and life expectations, thus having a negative impact on mental health (105). Moreover, relevant research shows that even with medical insurance and industrial injury insurance, high out-of-pocket expenses and economic insecurity will limit people’s access to mental health services (106). Therefore, women need to pay more attention to eliminating the negative mental health problems caused by economic insecurity in the process of accumulating financial capital.

### 5.2 Potential mediated associations between livelihood capital and mental health

Based on the empirical results of the mediation model, relative deprivation had a mediating impact on the pathway from livelihood capital to women’s mental health, supporting H2. While there has been a focus in research concerning differences in direct relationships between livelihood capital and women’s mental health, less attention has been paid to the mediating effect of relative deprivation. Therefore, we considered the mediating role of relative deprivation in the relationship between livelihood capital and women’s mental health. First, the study found that relative deprivation mediated the relationship between human capital and women’s mental health. This effect may stem from economic and social inequalities that marginalize women, contributing to mental health decline (107). Relevant studies show that older women are particularly vulnerable to social deprivation, which may be due to income reduction after retirement, changing social roles, and other factors that negatively affect their mental health (56). Additionally, women’s access to social resources, such as education and employment, is often more limited compared to men’s, placing them at a disadvantage in the workplace and social settings, which adversely affects their health (108). Second, social capital positively influenced women’s mental health through its effects on relative deprivation, which was consistent with other research findings. This could be because social capital enhances individual endowments and income, improving socioeconomic status and reducing social inequality, thereby promoting women’s mental health (109). Finally, the relationship between physical capital and women’s mental health was mediated by relative deprivation, possibly because communities with high levels of relative deprivation often feature crowded living conditions in family housing, leading to poorer social relationships among women and lower levels of mental health (110).

### 5.3 Livelihood capital and women’s mental health between urban and rural women

The results confirmed that the impact of livelihood capital on the mental health of urban and rural women varied, supporting H3. To start with, human capital had a stronger impact on the mental health of rural women, which was consistent with previous studies. This might be attributed that compared with women in urban areas, women living in rural areas receive poorer education level and medical care level, which led to lower happiness. Relevant research showed that higher education and access to medical experts can effectively prevent mental health problems (111). Second, social capital had a more significant influence on rural women’s mental health (112), such as social support, neighborhood relationships, community trust, and neighborhood mutual aid. Compared to urban women, rural women reported feelings of loss, which might be due to lower educational levels and the loss of their original social status in their hometown while working in urban areas, without an opportunity to regain an equivalent status level (113). In addition, numerous rural women migrated to urban areas for work. Compared to local urban residents, these rural women might be more likely to experience anxiety and depression because of their heightened sensitivity to interpersonal relationships and lower levels of community trust (114). Moreover, community cohesion was beneficial for the mental health of women. Besides, research has shown that it is difficult to establish rapport with local citizens after migrating to urban areas for work (114). Therefore, such women struggled to feel safe and secure in neighborhood interactions, which affected their mental health. Furthermore, this study suggested that physical capital did not affect urban women’s mental health but had a positive impact on rural women’s mental health, which differed from most previous study findings. This might be attributable to the limited variability in housing conditions among the urban participants, with most living in substandard living environments. Additionally, the small sample size of the urban participants made it difficult to identify associations with mental health in such a relatively small study population (115). Compared with urban areas, housing was mostly self-built in rural regions, with walls often constructed of mud and with strong unimpeded currents of air within houses. These conditions could adversely affected the physical health and sleep quality of residents, potentially leading to depression (101). In China, rural households were often multi-generational and large. Studies have shown that living with more people may increase the likelihood of developing depression (116). Additionally, several rural women who migrated to urban areas for work often lived in overcrowded and inadequately equipped housing, which affected their mental health. These factors may explain why the rural women placed greater importance on housing conditions. Interestingly, financial capital had a negative impact on urban women’s mental health, but it did not affect rural women. This may be because compared with rural areas, along with the increase of income and financial assets, the living, economic and social pressure in urban areas was generally greater. Relevant evidence suggested that living in urban communities with higher chronic stress was more likely to lead to cognitive, emotional, and social vulnerability in women (117).

This study established that the relationship between livelihood capital and rural women’s mental health was mediated by relative deprivation, whereas no mediating effect was observed in the model for urban women. This finding also supported H3. Relative deprivation was more related to perceived status differences among the residents. Rural women were more susceptible to social inequality and exclusion in their social milieu due to their rural resident status and did not have access to the same level of opportunities in terms of social welfare and education compared to urban women. Therefore, their mental health might be more vulnerable to the effects of relative deprivation (118). As rural women were likely to have lower levels of education and work ability, leading to a sense of relative deprivation, there might be more depression in rural women related to weaker human capital and a lower perception of their socioeconomic status (118). Furthermore, social capital affected rural women’s mental health through relative deprivation. This might be attributable to more limited social welfare support and security for rural women compared to urban women, making it harder for rural women to feel a sense of belonging in the community when living in urban areas. They may experience feelings of exclusion and isolation, leading to a sense of community deprivation, which in turn affected their mental health (119). Finally, relative deprivation played a mediating role in the relationship between physical capital and the mental health of rural women, which can be attributed to peer influence where rural women were more likely than urban women to compare their housing conditions with those of their neighbors. Such comparisons would highlight housing inequality, leading to a sense of social deprivation and a possible deterioration in mental health (120).

This study had several limitations. First of all, owing to limitations in the sample size, we excluded some urban samples with smaller numbers to prevent interference with our data conclusions. This resulted in a limited variety of provincial samples, making it difficult to fully analyze potential associations in women’s mental health in relation to specific provinces. Second, women’s mental health was influenced by multiple factors, some of which might not have been adequately considered, while others that we were aware of could not be considered in this study owing to data limitations. Therefore, this study provided only an initial analysis and summary of specific factors. Third, the conclusions might have time-specific relevance, as the impact of some factors on women’s mental health could change over time.

## 6 Conclusion

This study explored the relationship among livelihood capital, relative deprivation, and women’s mental health. The results showed that there was a positive relationship between women’s mental health, human capital, social capital, and physical capital, with increases in human capital, social capital, and physical capital, leading to improved women’s mental health. Besides, financial capital was negatively associated with women’s mental health. At the same time, relative deprivation played a mediating role in the impact of livelihood capital on women’s mental health. In addition, this study found that there were distinctive differences in terms of the effects of human, social, physical and financial capital on women’s mental health between urban and rural women. Human, social and physical capital had a stronger impact on the mental health of rural women than on urban women, while financial capital had a stronger influence on the mental health of urban women than on rural women. Moreover, livelihood capital indirectly affected rural women’s mental health through relative deprivation. However, this study found no indication that the relationship between livelihood capital and the mental health of urban women was mediated by relative hardship.

With regard to the social welfare implications of these findings and given the various critical challenges facing women’s mental health in both urban and rural areas, there is a need for targeted improvements in mental health services for women in different regions, especially in rural areas. Efforts should be focused on enhancing mental health support and providing resources to promote social equality. With respect to public service initiatives, implementing measures such as improving housing conditions and fostering better neighborhood relationships to create a more supportive environment for women’s mental health could help alleviate imbalances in addressing issues in women’s mental health and provide more comprehensive support and assistance. Moreover, it is crucial to promote mental health education by providing more extensive mental health guidance and educational opportunities, especially to women in rural areas. This could be achieved by organizing lectures, courses, or online resources. Additionally, it is important to build support networks by creating support groups, communities, or organizations that offer platforms for rural women to connect and support each other, which would help them share experiences, reduce stress, and foster social connections.

## Figures and Tables

**Figure A1:**
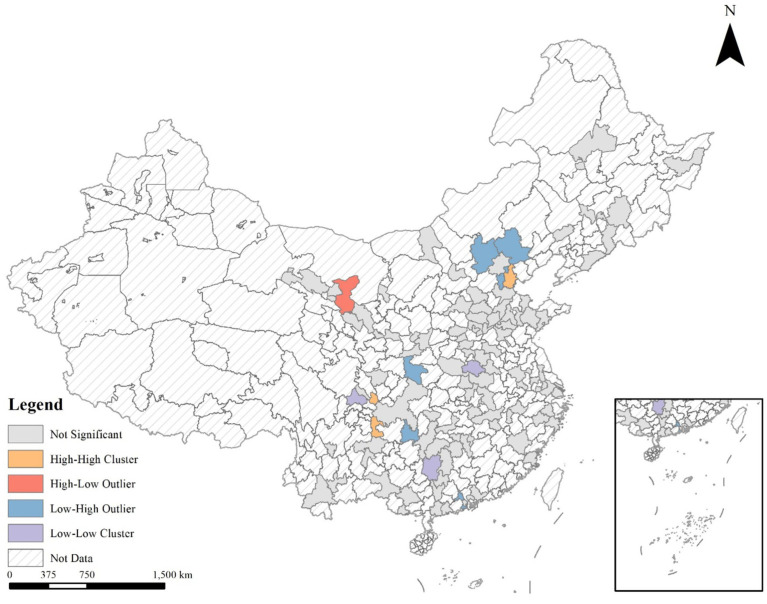
LISA cluster diagram of women’s mental health in China. Based on the Department of Natural Resources Standard Map Service website GS (2024)0650. Standard maps are produced with no modifications to the base map boundaries, same as below.

## Data Availability

The original contributions presented in the study are included in the article/supplementary material, further inquiries can be directed to the corresponding author.

## Appendix

Spatial distribution analysis of women’s mental health

Global and local spatial autocorrelation analyses was performed to detect spatial correlations between women’s mental health levels. Moran’s I statistic was used for the global spatiotemporal analysis to examine the presence or absence of spatial clusters in terms of women’s mental health in China. This study employed a global autocorrelation measure to describe and visualize the spatial distribution of women’s mental health based on the Moran’s I statistic, with data derived from the 2018 CLDS. The results demonstrated that the value of Moran’s I is 0.11 with a Z-score of 3.35 and a *p*-value below 0.001, which confirmed that women’s mental health had a significant spatial autocorrelation. Additionally, this research further used the local indicators of spatial association (LISA) map to detect the local autocorrelation (Figure A1). The high-high hot spots of women’s mental health were in Suining City, Luzhou City, and Tianjin City, while the low-low hot spots were found mainly in Chengdu, Zhumadian and Guilin City. And the low-high hot spots of women’s mental health were observed in northeastern Hebei Province, central and southern Guangdong Province, Ankang City and Tongren City, while high-low clustering was observed in Wuwei City. The LISA and Moran’ I data indicate that there were significant spatial variabilities of women’s mental health in China.
